# Potential role of the spleen in the development of arterial hypertension in humans

**DOI:** 10.1097/HJH.0000000000004115

**Published:** 2025-11-05

**Authors:** Matteo Nardin, Claudia Agabiti Rosei, Claudia Rossini, Fabio Bertacchini, Silvia Piantoni, Valeria Brami, Giulia Chiarini, Paolo Malerba, Niccolò Piacentini, Samantha Sartori, Silvia Ministrini, Antonella Anastasia, Mariella D’Adda, Enzo Porteri, Paolo Airò, Guido Alberto Massimo Tiberio, Giuseppe Rossi, Franco Franceschini, Damiano Rizzoni, Carolina De Ciuceis

**Affiliations:** a Division of Internal Medicine, Department of Medicine, ASST Spedali Civili di Brescia; b Division of Internal Medicine, Department of Clinical and Experimental Sciences, ASST Spedali Civili Brescia, University of Brescia; c Emergency Medicine, Department of Medicine, ASST Spedali Civili di Brescia; d Rheumatology and Clinical Immunology Unit, Department of Clinical and Experimental Sciences, ASST Spedali Civili and University of Brescia, Brescia, Italy; e The Zena and Michael A. Wiener Cardiovascular Institute, Icahn School of Medicine at Mount Sinai, New York, New York, USA; f Surgical Clinic, Department of Clinical and Experimental Sciences, University of Brescia, Brescia and ASST Spedali Civili di Brescia; g Hematology Division, Department of Oncology, ASST Spedali Civili di Brescia; h Division of Medicine, ASST Spedali Civili, Montichiari, Brescia, Italy

**Keywords:** humans, hypertension, lymphocytes, spleen, wall to lumen ratio

## Abstract

**Objectives:**

Immunity, particularly T lymphocytes, plays an important role in the development of arterial hypertension. Moreover, the so-called neuro-immune axis has been identified as a crucial crossroads, occurring in the spleen and involving placental growth factor as the principal mediator. However, no studies in humans have yet investigated the role of the spleen in hypertension and vascular damage.

**Methods:**

In this retrospective, case-control, single-blind study, we enrolled patients who had previously undergone elective splenectomy (cases) and subjects who had undergone elective cholecystectomy (controls). All subjects underwent 24-h ambulatory blood pressure monitoring, evaluation of retinal arteriole morphology by adaptive optics, capillary density assessment by video-capillaroscopy, arterial stiffness measurements, and analysis of T lymphocyte subpopulations by flow cytometry.

**Results:**

Fifty patients were included: 25 (50%) cases and 25 (50%) controls. No difference in hypertension prevalence (*P* = 0.39) or cumulative incidence (*P* = 0.79) of new diagnoses was detected. Splenectomized patients displayed lower 24-h (*P* = 0.024) and daytime (*P* = 0.011) diastolic blood pressure compared to cholecystectomized patients. Similar results were obtained for retinal structural parameters, capillary density, and arterial stiffness between the groups. A significant impact of splenectomy on the relationship between 24-h diastolic BP and wall cross-sectional area (*P*-interaction = 0.019) and forearm capillary density recruitment (*P*-interaction = 0.020) was found. A higher number and percentage of CD3^+^CD8^+^ T cells were observed in splenectomized patients compared to cholecystectomized patients (*P* = 0.009 and *P* = 0.001, respectively), although no differences in cytokine production patterns were detected.

**Conclusions:**

For the first time, our results support the role of the spleen in blood pressure control in humans. Further and larger studies are required to appropriately translate our findings into clinical practice.

## INTRODUCTION

Cardiovascular diseases are the leading cause of mortality worldwide, particularly in Western nations, with an increasing trend also observed in developing countries. Among the primary factors contributing to the heightened risk of cardiovascular diseases, high blood pressure, or hypertension, stands out as the most significant preventable risk factor [1]. A substantial body of scientific literature has demonstrated that measures to lower blood pressure can significantly reduce premature morbidity and mortality [2,3].

Despite these findings, achieving optimal blood pressure control remains a challenge. This shortfall can be partly attributed to the limited understanding of the underlying causes of essential hypertension [4], which is diagnosed in the vast majority of hypertensive patients. Recent evidence has highlighted the involvement of the immune system and neuro-immune interactions in the pathogenesis of hypertension. The first report suggesting a potential role of T lymphocytes in hypertension was provided by Shao *et al.* [5]. Subsequent investigations revealed that mice deficient in both T and B lymphocytes did not develop vascular damage in various experimental models of hypertension [6]. Furthermore, recent experimental studies have emphasized the protective role of regulatory T cells (Treg) [7]. However, the exact mechanisms underlying the interaction between the immune system and blood pressure regulation remain unclear.

One molecule that has garnered increasing interest is placental growth factor (PlGF). Known for its role as an angiogenic cytokine [8], experimental studies suggest that PlGF may mediate angiotensin II-induced hypertension, highlighting the spleen's pivotal role in the PlGF signaling pathway. Specifically, PlGF expression is induced during the prehypertensive phase of angiotensin II infusion in the stromal tissue of the spleen, particularly in the marginal zone of murine models [9].

The spleen plays a critical role in immune regulation, acting as a central hub where various immune components interact. These interactions within the spleen enable rapid sensing of challenges, as well as subsequent differentiation and migration of T cells [10]. In experimental models of angiotensin II-induced hypertension, it has been demonstrated that T cells found in target tissues, such as the kidneys and vasculature, originate in the spleen. In the same experimental context, splenectomy prevented T cell infiltration and blood pressure elevation, underscoring the spleen's essential role in modulating immune balance and hypertension [9].

The sympathetic nervous system also appears to be a key regulator of splenic immune function. The spleen is primarily innervated by noradrenergic efferent fibers originating from the superior mesenteric celiac ganglion [11]. These fibers travel along the splenic artery and penetrate into the white pulp [12]. Earlier research suggested a connection between brain-mediated immune responses occurring in the spleen and resulting blood pressure variations [13]. Subsequent studies have shown that, similar to what is observed in the renal system, angiotensin II increases the sympathetic release of noradrenaline in the spleen [14]. Moreover, selective denervation of sympathetic innervation to the spleen has been shown to protect against blood pressure increases and immune activation in response to angiotensin II in murine models [14].

Based on this preclinical evidence, we aimed to explore, in a case–control study, the potential impact of the spleen on arterial blood pressure, microcirculation, arterial stiffness, and T lymphocyte subpopulations in patients who underwent splenectomy at least five years prior, compared to controls treated with cholecystectomy.

## METHODS

This was a retrospective, case-control, single-blinded study. For the case group, eligible patients were those who underwent elective splenectomy for immune thrombocytopenic purpura or marginal lymphoma between 1998 and 2010 at the General Surgery Unit and were under regular follow-up at the Hematology Unit of ASST Spedali Civili, Brescia, Italy.

For the control group, we enrolled patients who underwent elective cholecystectomy during the same period at the 3rd General Surgery Division of Spedali Civili, Brescia. Control group patients were randomly selected and matched by age and sex to the case group subjects.

For both cases and controls, the inclusion criteria were age between 20 and 70 years at the time of the surgical intervention and signed consent to be enrolled in the study. Exclusion criteria included the presence of any of the following conditions: active cancer, autoimmune diseases, conditions leading to immune system impairment (e.g., human immunodeficiency virus infection), or ongoing treatment with immunomodulatory or antiviral drugs.

Lists of eligible patients were provided by the Hematology and General Surgery Units. Patients were contacted by phone, and an outpatient visit was scheduled. Written informed consent was obtained from all enrolled participants. During the visit, data was collected on medical history, current treatments, and recent laboratory results (no older than two months).

Dedicated personnel, who were aware of each patient's group assignment, handled phone calls and collected anamnestic data. The following assessments were performed: video-capillaroscopy, evaluation of retinal arteriolar morphology using adaptive optics (AO), and 24-h ambulatory blood pressure monitoring (ABPM). Venous blood samples were collected to measure the percentage of pitted erythrocytes as an indicator of splenic function [15]. All physicians and biotechnologists conducting these procedures were blinded to the patient group assignments. All collected data were entered into a dedicated, password-protected database. This study was approved by the local ethics committee and adhered to the principles outlined in the Declaration of Helsinki.

### Evaluation of splenic function

Splenic function was assessed by counting pitted red blood cells (i.e., red blood cells with membrane abnormalities visible under interference phase microscopy, referred to as “pits”). This method is recognized for its high accuracy and reproducibility [16]. We followed a validated protocol from prior studies [17]. Briefly, a drop of fresh venous blood was mixed with 0.5 ml of 1% buffered-glutaraldehyde solution at pH 7.4. Wet preparations were examined under a direct-interference contrast microscope (Olympus BX50, Hamburg, Germany) equipped with Nomarski optics at a magnification of 1000×. An independent observer, blinded to the patients’ diagnoses, analyzed 1000 red blood cells. The percentage of pitted red blood cells was calculated and used as a measure of splenic function. Based on existing literature [18–20], an upper limit of 10% pitted red blood cells was adopted to define normal spleen function, thereby maximizing the specificity for detecting asplenism.

### Ambulatory blood pressure monitoring

Twenty-four-hour ABPM was performed in all patients (Oscar 2 Ambulatory Blood Pressure Monitor from SunTech Medical). The device's interpretative software was used to estimate central blood pressure values and the augmentation index (AIx), standardized at 75 bpm (AIx@75) [21]. Only patients with valid ABPM recordings obtained at the time of study entry were included in the analysis. Valid recordings were defined as those meeting the criteria of at least 70% of the expected number of readings, with a minimum of 20 valid readings during daytime hours and 7 during nighttime hours, as recommended by current guidelines [1]. The ABPM device was programmed to measure blood pressure at intervals of at least 15 min during the daytime and at least 30 min during the nighttime. Monitoring began between 9 : 00 a.m. and 12 : 00 a.m. to ensure standardized data collection and facilitate comparisons. The monitoring cuff was placed on the nondominant arm, and patients were instructed to keep their arm still and avoid movement during measurements. Participants were allowed to carry out their usual daily activities, with the exception of strenuous exercise and showering.

### Evaluation of capillary density

Skin capillary density was assessed using video-capillaroscopy before and after venous congestion, as previously described [22,23]. Briefly, after a resting period in a seated position in a quiet, temperature-controlled room, capillaries from the nailfold and the dorsum of the fourth finger on the nondominant hand, as well as from the forearm of the same arm, were visualized using an epi-illuminated microscope equipped with a 100-W mercury vapor lamp.

Images were obtained using video-microscopy (Videocap 3.0 D1 200; DS Medica, Milan, Italy) at a final magnification of 200×. Capillaries were imaged under baseline conditions (basal capillary density) and after venous congestion (total capillary density) to visualize previously excluded functional capillaries. Venous congestion was induced by inflating a miniature blood pressure cuff placed at the base of the fourth finger on the nondominant hand to 60 mmHg for 2 min.

Capillary density, defined as the number of capillaries per square millimeter of the microscopic field, was manually counted. Only the first row of nailfold capillaries was considered. Recruitment in each area was calculated as the percentage increase in capillary density between total and basal conditions. Capillary density assessments were performed independently by two operators, and the results were averaged.

### Evaluation of retinal arteriolar morphology

AO is an advanced, noninvasive optoelectronic method that provides high-resolution qualitative and quantitative measurements of microvascular morphology in the human retina at near-histological scale [24]. Fundus images were captured using a commercially available flood-illumination AO camera (Rtx-1; Imagine Eyes, Orsay, France). The Rtx-1 AO camera uses a 750-nm super-luminescent diode light source and a closed-loop system to measure and correct wavefront aberrations [25]. A 4 × 4 fundus area (approximately 1.2 × 1.2 mm in emmetropic eyes) was illuminated at 840 nm by a temporally low-coherence light-emitting diode. A stack of 40 fundus images was acquired over 4 s by the camera. Pupil dilation was not required. After a 5-min rest, the patient was positioned on the chin rest. A live video image of the pupil was used to align the incident light, while a live display of the AO-corrected fundus image allowed adjustments to brightness, contrast, and focus. The gaze target was oriented to capture the superotemporal artery of the right eye. The segment of interest was free from bifurcations, at least 250 μm in length, and had an inner diameter of at least 50 μm. Areas with focal arterial nicking or arteriovenous crossings were excluded. Specialized image processing software was used to measure the inner and outer diameters of the vessel [25]. Wall thickness was defined as wall thickness = (outer diameter – inner diameter)/2, total wall cross-sectional area (WCSA) as


$$WCSA=\pi \cdot \left[{\left(\frac{outer\;diamenter}{2}\right)}^{2}-{\left(\frac{inner\;diamenter}{2}\right)}^{2}\right]$$

and the ratio of total parietal thickness over the lumen diameter averaged along 250-μm length defined the wall-to-lumen ration (WLR) [26]. The average values and SD were obtained by averaging 30 measurements.

### Pulse wave velocity

Arterial stiffness was assessed by measuring pulse wave velocity (PWV), considered the gold standard for this evaluation [1]. Measurements were taken with patients in a supine position, with the neck slightly hyperextended, following 15 min of rest. PWV was determined at the carotid and femoral arteries using the foot-to-foot velocity method [27]. Waveforms were obtained transcutaneously at the right common carotid artery and the right femoral artery, and the time delay (transit time) between the feet of the two waveforms was measured using a dedicated device (Complior SP: Artech Medical, Pantin, France). The distance traveled by the pulse waves was estimated as the direct distance between the two recording sites (carotid–femoral distance). PWV was calculated as the ratio of the carotid-femoral distance to the transit time, with an adjustment factor of 0.80 applied as previously recommended [28]. A PWV >10 m/s was considered indicative of macrovascular target organ damage^1^. Only patients with high-quality PWV signals, defined as a standard deviation of less than 5% across at least 10 cardiac cycles, were included in the analysis.

### Evaluation of circulating T lymphocyte subpopulations and cytokines expression

Circulating T-lymphocytes subpopulations were evaluated by flow cytometry. The expression of Interleukin-17 (IL-17) and interferon-γ (INF-γ) was also performed in overall T-cells population and among two T-cells subgroup, CD3^++^CD4^+^ and CD3^+^CD8^+^. Details are provided in Supplementary material.

### Statistical analysis

Considering a mean systolic blood pressure of 120 mmHg, with a standard deviation of 8 mmHg at 24-h ABPM in the general population, a sample size of 40 patients per group provides a power of 80% for detecting a 5 mmHg difference at an α-error of 0.05. Patients were analyzed according to their assigned group, splenectomy or cholecystectomy. Continuous data were evaluated using the Shapiro–Wilk test to confirm normal distribution. Analysis of variance was performed if normal distribution was confirmed; otherwise, nonparametric tests were used. Data are presented as median and interquartile range (IQR). Fisher's exact test was used for categorical variables.

An UpSet plot was generated for quantitative visualization of the antihypertensive drugs combinations in patients underwent splenectomy and cholecystectomy, separately.

Cumulative incidence of hypertension was assessed using the Kaplan–Meier method and compared between groups with the log-rank test among nonhypertensive patients at the time of surgical intervention. Proportional hazards Cox regression was used to evaluate the association between splenectomy and new diagnosis of hypertension. The multivariate model included the patients’ age at the time of surgical intervention.

Linear regression analyses were performed to evaluate the relationship between ABPM values, video-capillaroscopy, AO measures and PWV. Models were also constructed to evaluate the interaction between regression results and groups. Combined scatter plot was generated for visualization of continuous variables and interaction between groups. A p-value <0.05 was considered statistically significant.

## RESULTS

A total of 50 patients were included in the study, with 25 (50%) undergoing splenectomy and 25 (50%) undergoing cholecystectomy. The study flow diagram is presented in Figure S1, Supplemental Digital Content. Asplenism in the splenectomy group was confirmed through the evaluation of pitted red blood cells: all patients in the splenectomy group exhibited pitted erythrocyte percentages greater than 10%, whereas all subjects in the cholecystectomy group had values below 5% (Figure S2, Supplemental Digital Content).

The main baseline characteristics of the two groups are summarized in Table 1. A higher prevalence of hepatic comorbidities, primarily hepatic steatosis, was observed among patients in the cholecystectomy group. As expected, higher white blood cell and platelet counts were noted in patients who had undergone splenectomy.

**TABLE 1:** Baseline differences in two groups

Characteristics	Splenectomy (*n* = 25)	Cholecystectomy (*n* = 25)	*P*-value
Age (years)	66.73 [59.95–70.13]	66.13 [63.92–67.44]	0.78
Male sex (%)	36.0	36.0	1
BMI (kg/m^2^)	24.56 [23.12–28.52]	26.29 [23.44–27.55]	0.16
Hypercholesterolemia (%)	32.0	44.0	0.56
Diabetes mellitus (%)	20.0	8.0	0.42
Chronic kidney disease (%)	12.0	0.0	0.24
Familiarity for CAD (%)	32.0	28.0	0.76
Previous MI (%)	8.0	8.0	1
Previous CVE (%)	0.0	12.0	0.24
Carotid atherosclerosis (%)	12.0	16.0	0.68
Smoking			0.22
Never (%)	52.0	60.0	
Active (%)	20.0	4.0	
Former (%)	28.0	36.0	
Alcohol assumption			0.52
Never (%)	52.0	36.0	
Occasionally (%)	24.0	36.0	
Daily (%)	24.0	28.0	
Coffee (*n*/die)	2 [1–3]	2 [1–2]	0.14
Comorbidities			
Cardiovascular (%)	8.0	24.0	0.25
Respiratory (%)	20.0	40	0.19
Endocrine (%)	28.0	24.0	0.74
Gastro-enteric (%)	36.0	44.0	0.77
Hepatic (%)	4.0	32.0	0.023
Office systolic BP (mmHg)	127 [120–135]	130 [120–130]	0.47
Office diastolic BP (mmHg)	80 [72–81]	80 [75–85]	0.40
Office heart rate (bpm)	70 [68–73]	68 [65–73]	0.81
WBC (^a^10^3^/μl) [normal value: 4.00–10.80]	8.50 [7.20–12.95]	5.80 [5.25–6.24]	<0.001
RBC (^a^10^6^/μl) [normal value: 4.00–5.20; 4.50–5.50]^a^	4.54 [4.26–4.84]	4.82 4.60–4.97]	0.035
Haemoglobin (g/dl) [normal value: 12–16; 13–18]^a^	13.7 [12.9–14.5]	14.5 [13.8–15.0]	0.13
Hematocrit (%) [normal value: 37.0–47.0; 42.0–52.0]^a^	42.2 [40.0–45.1]	43.5 [42.3–45.0]	0.14
Platelets (^a^10^3^/μl) [normal value: 130–400]	322 [270–380]	213 [170–264]	<0.001
Glycaemia (mg/dl) [normal value: 70.0–100.0]	92.5 [84.5–108.5]	91 [87–108]	0.87
Creatinine (mg/dl) [normal value:0.50–0.95; 0.70–1.16]^a^	0.74 [0.67–0.89]	0.80 [0.70–1.01]	0.43
Total cholesterol (mg/dl) [normal value: 120–200]	193 [166–229]	189 [154–229]	0.89
HDL cholesterol (mg/dl) [normal value: >46; >38]^a^	58 [44–79]	55 [47–75]	0.65
Triglycerides (mg/dl) [normal value: <150]	89 [54–113]	87 [74–147]	0.99
AST (U/l) [normal value: 9–31; 13–51]^a^	20 [19–21]	23 [20–31]	0.065
ALT (U/l) [normal value: 11–29; 15–47]^a^	22 [16–29]	25 [19–44]	0.19

ALT, alanine aminotransferase; AST, aspartate aminotransferase; BMI, body mass index; BP, blood pressure; CAD, coronary artery disease; CVE, cerebrovascular events; HDL, high density lipoprotein; MI, myocardial infarction; RBC, red blood cells; WBC, white blood cells.

a The first normal value is for female patients; the second normal value is for males.

### Hypertension

**FIGURE 1:**
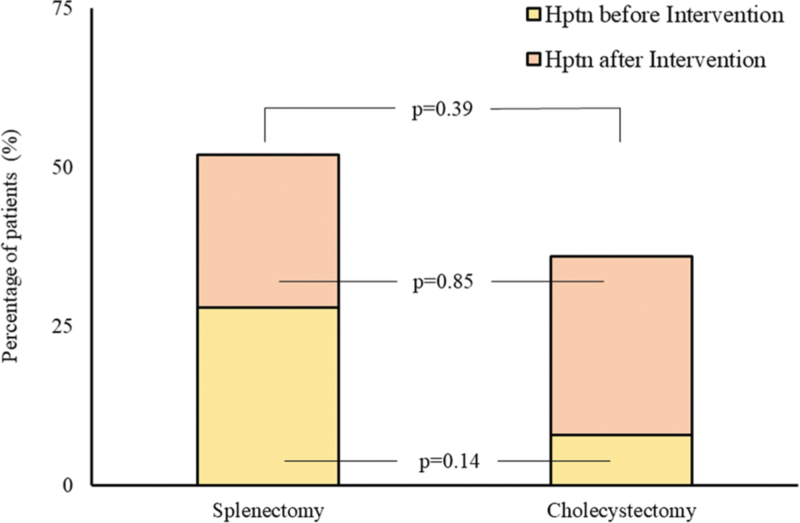
Percentage of hypertensive patients. Bar graph showing the percentage of patients that had a diagnosis of hypertension before the surgical intervention (green portion) and percentage of patients who received a diagnosis of hypertension between surgical intervention and follow-up visit (red portion).

Figure 1 illustrates the percentages of patients diagnosed with hypertension at the time of surgical intervention (28% in splenectomized patients vs. 8% in cholecystectomized patients; *P* = 0.14) and the incidence of new hypertension diagnoses after surgery (24% vs. 28%, respectively; *P* = 0.85), resulting in an overall prevalence of 52% among splenectomized subjects compared to 36% in controls (*P* = 0.39). ABPM values, including brachial and central blood pressure averages, are detailed in Table 2 for both groups across different time periods. While no significant differences were observed in 24-h average systolic blood pressure, splenectomized subjects exhibited significantly lower 24-h average diastolic and mean blood pressure values compared to cholecystectomized subjects. This difference was primarily driven by variations in daytime averages. Similar findings were noted for central 24-h and daytime average diastolic blood pressure. Antihypertensive drug combinations for cases and controls are presented in Figure S3, Supplemental Digital Content. For patients without a prior diagnosis of hypertension at the time of surgical intervention, the cumulative incidence of new hypertension diagnoses did not differ significantly between groups (*P* = 0.79) (Figure S4, Supplemental Digital Content). Splenectomy was not significantly associated with an increased risk of developing hypertension (hazard ratio [HR] [95% confidence interval (CI)]: 1.17 [0.37–3.71]; *P* = 0.79). This result remained unchanged after adjusting for patients’ age at the time of intervention (adjusted HR [95% CI]: 1.29 [0.38–4.39]; *P* = 0.69).

**TABLE 2:** Ambulatory blood pressure monitoring results

ABPM measures	Splenectomy (*n* = 25)	Cholecystectomy (*n* = 25)	*P*-value
Brachial measurement			
24h			
Systolic BP (mmHg) [normal value: <130]	117 [109–125]	119 [115–125]	0.78
Diastolic BP (mmHg) [normal value: <80]	65 [63–72]	72 [69–76]	0.024
Mean BP (mmHg)	85 [78–88]	90 [85–92]	0.024
Daytime			
Systolic BP (mmHg) [normal value: <135]	121 [112–127]	124 [117–128]	0.78
Diastolic BP (mmHg) [normal value: <85]	68 [65–74]	75 [70–79]	0.011
Mean BP (mmHg)	87 82–90]	92 [86–97]	0.047
Night-time			
Systolic BP (mmHg) [normal value: <120]	114 [101–119]	108 [103–118]	0.77
Diastolic BP (mmHg) [normal value: <70]	58 [53–67]	65 [60–69]	0.26
Mean BP (mmHg)	74 [70–87]	79 [74–84]	0.39
Central measurement			
24h			
Systolic BP (mmHg)	110 [103–116]	111 [108–117]	0.78
Diastolic BP (mmHg)	67 [64–73]	73 [71–77]	0.011
AIx@75 (%)	34 [28–44]	40 [32–47]	0.57
Daytime			
Systolic BP (mmHg)	110 [107–117]	115 [110–117]	0.40
Diastolic BP (mmHg)	69 [66–76]	77 [71–81]	0.011
AIx@75 (%)	35 [27–43]	41 [31–47]	0.39
Night-time			
Systolic BP (mmHg)	108 [97–112]	102 [97–113]	0.24
Diastolic BP (mmHg)	60 [56–69]	65 [61–70]	0.15
AIx@75 (%)	38 [31–45]	40 [35–47]	0.77

ABPM, ambulatory blood pressure monitoring; AIx@75, augmentation index standardized at hear rate of 75 beat per minute; BP, blood pressure.

### Microcirculation and arterial stiffness

Parameters of retinal microcirculation evaluated through AO, video-capillaroscopy, and arterial stiffness assessments are presented in Table 3. No significant differences were observed between groups, except for a trend toward higher WCSA in splenectomized patients compared to those who underwent cholecystectomy.

**TABLE 3:** Adaptive optics, video-capillaroscopy, and arteria stiffness assessment

Variables	Splenectomy (*n* = 25)	Cholecystectomy (*n* = 25)	*P*-value
Adaptive optics			
WCSA, μm^2^	4868 [3820–5411]	4027 [3314–4838]	0.087
WLR, absolute	0.28 [0.26–0.32]	0.29 [0.27–0.32]	0.35
Video-capillaroscopy			
*Periungual*			
Basal capillary density, *n*	8 [7–9]	9 [8–10]	0.13
Recruitment, %	0	0	-
*Dorsal finger*			
Basal capillary density, *n*	80 [64–94]	75 [62–94]	0.31
Recruitment, %	6 [0–9.8]	0 [0–20]	0.31
*Forearm*			
Basal capillary density, *n*	53 [45–59]	56 [45–67]	0.30
Recruitment, %	8 [0–30]	14 [4–20]	0.46
Arterial stiffness			
PWV crude, m/s	11.21 [9.76–12.84]	11.07 [9.88–13.13]	0.89
Adjusted PWV, m/s	8.97 [7.81–10.27]	8.86 [7.90–10.50]	0.89
Adjusted PWV >10 m/s, %	40.0	29.4	0.53

PWV, pulse wave velocity; WCSA, wall cross-sectional area; WLR, wall-to-lumen ratio.

In the overall cohort, no significant associations were identified between 24-h average BP values obtained via ABPM and microcirculation or arterial stiffness parameters (Table S1, Supplemental Digital Content).

**FIGURE 2:**
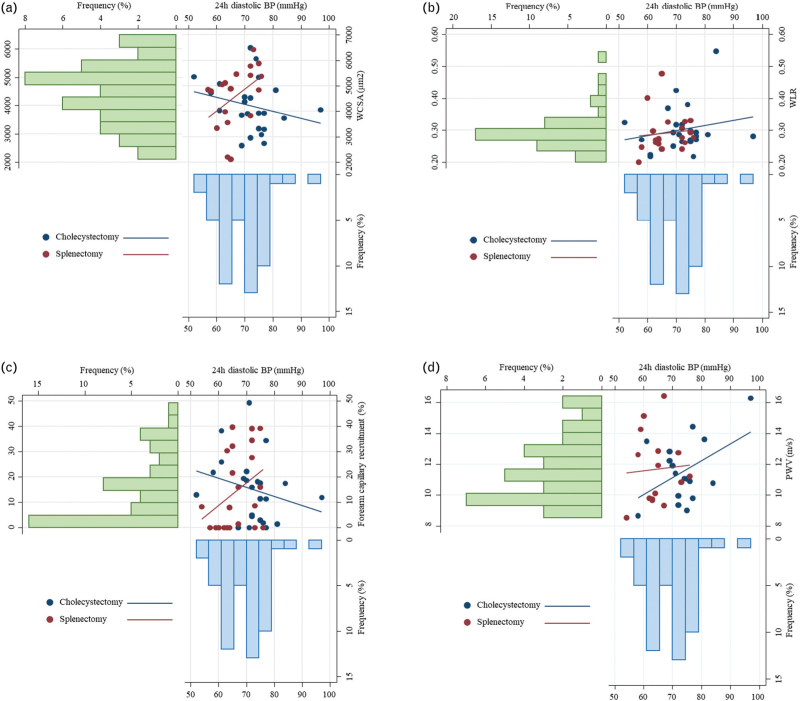
Relationship between 24-h diastolic blood pressure and micro- and macro-circulation parameters in cases and controls. Combined scatter plots and bar graphs showing the relation between 24-h (24 h) diastolic blood pressure (BP) and wall cross-sectional area (WCSA) (panel a), wall-to-lumen ratio (WLR) (panel b), forearm capillary recruitment (panel c) and pulse wave velocity (PWV) (panel d) in patients underwent splenectomy (dark red) and cholecystectomy (dark blue). Bar graph on top left (light green) shows the distribution of WCSA, WLR, forearm capillary recruitment and PWV values across the entire cohort, respectively in panel A, B, C and D; while bar graph on bottom right (light blue) represents the distribution of 24 h diastolic BP across the entire cohort in all panels.

However, distinct relationships were noted between WCSA and 24-h diastolic BP when assessed separately in splenectomized and cholecystectomized patients (*P*-_interaction_ = 0.019) (Fig. 2). Similarly, differences between the groups were observed in the relationship between forearm capillary recruitment and 24-h diastolic BP (*P*-_interaction_ = 0.020). In contrast, the relationships between WLR and PWV with 24-h diastolic BP remained consistent across both splenectomized and cholecystectomized patients.

### T-lymphocytes subpopulations and cytokine expression

The distribution of main T lymphocytes subpopulations is displayed in Table 4. Despite no difference in percentage, absolute number of T cells was higher among patients undergone splenectomy than cholecystectomized ones (*P* = 0.008), driven by overall higher number of lymphocytes. Splenectomized subjects had a significant higher number of CD3^+^CD8^+^ T cells, both absolutely and in percentage. However, number and percentage of T cells producing interferon-γ and Interluekin-17 were similar in the two groups across all T cells subpopulations.

**TABLE 4:** Circulating T-lymphocyte subpopulations in the examined population

Cells subgroups	Splenectomy (*n* = 25)	Cholecystectomy (*n* = 25)	*P*-value
CD3^+^			
T cells, %^a^	44.8 [33–6–62.0]	50.5 [38.5–50.5]	0.259
T cells, *n* (/μl)	1672 [955–2311]	1050 [719–1284]	0.008
INF-γ^+^ T cells, %^b^	1.39 [0.07–8.18]	1.78 [0.08–5.12]	0.670
INF-γ+ T cells, *n* (/μl)	27.2 [2.0–178.8]	6.2 [0.5–80.7]	0.301
IL-17+ T cells, %^b^	0.05 [0.02–0.15]	0.02 [0.002–0.18]	0.991
IL-17+ T cells, *n* (/μl)	0.91 [0.07–8.18]	0.5 [0.0–2.1]	0.576
CD3^+^CD4^+^			
T cells, %^b^	44.5 [38.1–55.4]	52.8 [41.9–62.4]	0.061
T cells, *n* (/μl)	629 [454–1208]	491 [360–712]	0.074
INF-γ+ T cells, %^c^	1.32 [0.0–6.65]	1.73 [0.08–3.98]	0.801
INF-γ+ T cells, *n* (/μl)	13.11 [0.0–51.65]	1.52 [0.31–35.95]	0.724
IL-17+ T cells, %^c^	0.03 [0.0–0.12]	0.02 [0.0–0.18]	0.655
IL-17+ T cells, *n* (/μl)	0.19 [0.0–2.72]	0.14 [0.0–1.09]	0.563
CD3^+^CD8^+^			
T cells, %^b^	40.6 [33.6–53.8]	34.8 [26.1–40.9]	0.009
T cells, *n* (/μl)	745 [316–1167]	320 [229–487]	0.001
INF-γ+ T cells, %^d^	0.25 [0.04–8.80]	1.82 [0.01–6.27]	0.874
INF-γ+ T cells, *n* (/μl)	2.81 [0.29–77.46]	1.49 [0–20.43]	0.373
IL-17+ T cells, %^d^	0.0 [0.0–0.05]	0.0 [0.0–0.09]	0.583
IL-17+ T cells, *n* (/μl)	0.0 [0.0–0.65]	0.0 [0.0–0.37]	0.678

CD, cluster of differentiation; IL-17, interleukin-17; INF-γ, interferon-γ.

a Percentage of total lymphocytes.

b Percentage of CD3^+^ T cells.

c Percentage of CD3^+^CD4^+^ T cells.

d Percentage of CD3^+^CD8^+^ T cells.

## DISCUSSION

This study represents the first investigation in humans of the role played by the spleen in the development and progression of hypertension. The main findings can be summarized as follows:

1. No significant differences in hypertension prevalence, nor new diagnosis of hypertension were appreciated between patients who underwent splenectomy and those who underwent cholecystectomy. The 24-h systolic BP and the majority of ABPM measures were similar in the two groups.
2. Significant lower values of 24-h diastolic and mean BP were detected in splenectomy group, whose the differences in daytime measures have mostly contributed.
3. The absence of spleen significantly impacted the relationship between 24-h diastolic blood pressure and WCSA and forearm capillary recruitment, whilst WLR and arterial stiffness.
4. An increased number of CD3^+^CD8^+^ T cells were detected among splenectomized patients compared to the controls.

Hypertension remains a complex disorder, and despite extensive research, its underlying mechanisms are not fully understood. Recent evidence suggests that the brain-to-spleen axis, which involves adaptive immunity, plays a crucial role in the development and progression of hypertension [9,29].

Prior studies have emphasized that the sympathetic nervous system is necessary for the activation of the immune system in the spleen, mediating its effects on BP through PlGF. While PlGF has been extensively studied in pregnant women due to its role in preeclampsia [30], its clinical significance in nonpregnant adults has often been overlooked due to negligible peripheral blood levels. However, PlGF has emerged as a key cofactor in immune activation, facilitating the interaction between innate and adaptive immunity through CD86 expression [9].

Lymphocytes, particularly the imbalance between Treg and Th17 cells, have been identified as essential mediators of hypertension, promoting pro-inflammatory and pro-hypertensive states [31]. Despite the decrease in total lymphocytes count, unbalancing the ratio of Treg and Th17 leads to pro-inflammatory and pro-hypertensive conditions [32,33]. Wang *et al.* provided further evidence regarding the central role of spleen in immune-mediated cardiovascular damage: they reported greater monocyte mobilization from the spleen and consequent higher macrophages recruitment into the heart and aorta after infusion of AngII in murine model [34]. Conversely, splenectomy and telmisartan reduced the recruitment into the heart, potentially ameliorating the AngII induced cardiac fibrosis and BP controls [34]. Despite these findings, controversies persist regarding the precise role of the spleen and the nerves in regulating BP. At the molecular level, PlGF appears to mediate the neuro-immune interaction between the sympathetic nervous system and the spleen, playing a pivotal role in lymphocyte activation [13].

In our study, patients who underwent splenectomy for immune thrombocytopenic purpura or marginal lymphoma were compared to patients who underwent elective cholecystectomy. This approach aimed to minimize the confounding effects of major abdominal surgery: both splenectomy and cholecystectomy are major abdominal surgeries that may lead to systemic physiological changes. Using cholecystectomy patients as controls allows for matching the potential effects of surgery and hospitalization, such as stress responses, perioperative inflammation, and recovery timelines. Therefore, differences observed between the two groups can more likely be attributed to the absence of the spleen rather than the effects of surgery itself [35]. The main finding was that splenectomized patients exhibited lower diastolic BP values compared to controls, with significant differences observed in both 24-h and daytime diastolic BP values. Despite the fact that diastolic values in both groups are within the normal range and, thus, do not independently impact cardiovascular risk [36], we agree with prior investigators on the complex interplay between diastolic blood pressure and the multiple mechanisms involved in its regulation [37,38]. Although these values per se are not independent predictors of adverse cardiovascular events, the observed differences support a potential role of the spleen in regulating blood pressure. Importantly, we did not match cases and controls based on preexisting hypertension; however, there were no significant differences in hypertension prevalence, prior diagnoses, or chronic treatments between the groups. The incidence of new hypertension diagnoses also did not differ between the two groups, even after adjusting for patients’ age at the time of surgery.

Microcirculatory assessments revealed a trend for lower WCSA among controls, while video-capillaroscopy did not show notable differences. A significant relationship was observed between WCSA values and 24-h average diastolic BP, with a direct correlation between WCSA and BP values in the splenectomy group. This suggests potential hypertrophic remodeling in response to changes in BP [39]. Of course it is possible that impairment of neuro-immune axis driven by spleen removal aggravates a preexisting organ damage, but unfortunately the microcirculation assessment preintervention was not performed, neither available for the majority of patients. However, due to the lack of preintervention microcirculation assessments, the causal link between splenectomy and microcirculatory changes remains speculative. Similarly, a direct relationship was observed between forearm capillary recruitment and BP values, but no significant differences were found in arterial stiffness as assessed by PWV.

There are several potential reasons for the discrepancies between our results and those observed in animal models. Firstly, nearly 30% of splenectomized patients in our study were already hypertensive at baseline, which may have limited the number of individuals who developed new hypertension after surgery. Moreover, as previously reported by Perrotta *et al.*, the spleen is not the sole component involved in the neuro-immune pathways that regulate BP [40]. In humans, the sympathetic nervous system may interact with other lymphoid organs in ways that are not captured in murine models. While the spleen plays a central role, it is possible that the absence of the spleen in humans does not fully mimic the immune and neurogenic interactions seen in mice.

In experimental models, the ablation of the neuro-splenic axis has been shown to have no effect under basal, nonpathological conditions, but it can protect against hypertension and related target organ damage [9,29]. This suggests that the neuro-splenic axis plays a protective role in hypertension, but its absence may not have the same effect in humans due to differences in immune system modulation and compensation by other organs.

To further investigate the potential mechanisms involved, we analyzed circulating T-lymphocyte subpopulations in both groups. We found an increased number of CD3^+^CD8^+^ T cells in splenectomized patients compared to controls. Additionally, we assessed cytokine production in response to specific stimuli and found no significant differences in interferon gamma (INF-γ) or interleukin (IL)-17 production between the two groups. However, other studies, such as those by Carnevale *et al.*, have shown that bioelectronic stimulation of the celiac vagus nerve can drive a selective egress of CD8^+^ T cells from the spleen [39]. As underlined by Carnevale D. [41], proofs of immunity involvement in hypertension imply an updating of role usually considered for sympathetic nervous system, from mere hemodynamic regulation to even modulation of immune response. Moreover the bidirectional brain–bone marrow axis and the gut-cardiac-immune axis are emerging pathways deeply involving and influencing human homeostasis, potentially contributing to blood pressure regulation [42–44].

Future steps of course will consider the introduction of therapies targeting the immune system in essential hypertension. However, careful consideration must be given to the risk/benefit ratio of immune-modulating therapies, given the potential side effects. The relationship between autoimmune diseases and hypertension has sparked interest in investigating whether immune-targeting therapies could affect hypertension as a comorbidity of autoimmune diseases, though results have been mixed [45,46]. Some immunosuppressant drugs have been clearly demonstrated to lower BP and hypertensive mediated organ damage [47,48].

Clinical evidence on this topic is constituted mostly by mechanistic association between hypertension and immune disorders. Basic science experiments improved our knowledge on neuro-immune signaling pathway, including mediators and organs involved, and hypertensive effects. Answers resultant by animal models, should lead the design of future powered trials to confirm the existence of same connections in humans and estimate their magnitude.

## LIMITATIONS

Our study has several limitations. First, our analyses were underpowered due to the lower-than-planned number of patients enrolled. The limited number of patients can be attributed to difficulties in retrospectively identifying individuals who had undergone splenectomy more than 10 years ago, are still alive, and remain under follow-up. Despite identifying a theoretically sufficient number of patients, various factors limited enrollment, as shown in Figure S1, Supplemental Digital Content. Second, ABPM and microcirculation data were not available at the time of the surgical intervention in either the splenectomy or cholecystectomy groups. However, the technology available at the time of the surgeries had lower accuracy than contemporary devices, which may have affected any potential comparisons. Third, the case-control design limits the external validation of our results, despite is the only available, to date, given the novelty of basic science findings and the long follow-up required to evaluate any potential protective roles driven by splenectomy. Fourth, we have not measured the levels of PlGF in our cases and controls that would have provided valuable information in overall interpretation. Data on lymphocyte sub-populations would have led to a more comprehensive view of the impact of splenectomy on the immune system and, consequently, on its relationship with hypertension.

## CONCLUSION

Our investigation represents the first study in humans to explore the impact of the spleen on arterial hypertension and vascular damage. Diastolic BP was found to be significantly lower in splenectomized patients, and the excision of the spleen appears to influence the relationship between WCSA, forearm capillary density, and 24-h diastolic BP. Preliminary results regarding T lymphocytes suggest an impact on immune cells involved in arterial hypertension. Further and larger studies are needed to appropriately translate our findings into clinical practice.

## ACKNOWLEDGEMENTS

The authors thank Elena Ostoni, biotechnologist, for excellent technical help.

Disclosures: No potential competing interest was reported by the authors.

### Conflicts of interest

There are no conflicts of interest.

## Supplementary Material

Supplemental Digital Content
