# Supplementary material for: Potential role of the spleen in the development of arterial hypertension in humans
Source: J Hypertens. 2025 Nov 5;44(1):71–80. doi: 10.1097/HJH.0000000000004115 (PMC12672046; doi:10.1097/HJH.0000000000004115)
Supplement: Supplemental Digital Content [file jhype-44-071-s001.docx]

**Supplementary material**

**Potential Role of the Spleen in the Development of Arterial Hypertension in Humans**

**Table of contents**:

- **Supplementary methods**: Evaluation of circulating T lymphocyte subpopulations and cytokines expression
- **Figure S1**. Study flow diagram
- **Figure S2**. Red blood cells in cases and controls
- **Figure S3**. Combinations of anti-hypertensive drugs
- **Figure S4**. Cumulative incidence of new diagnosis of hypertension
- **Table S1**. Linear regressions between wall cross-sectional area, wall-to-lumen ratio, dorsal and forearm capillary recruitment, pulse wave velocity and main values provided by ambulatory blood pressure monitoring

Supplementary methods: Evaluation of circulating T lymphocyte subpopulations and cytokines expression

*Evaluation of circulating T lymphocyte phenotype*

Peripheral blood T-cell phenotypic characterization was performed by flow cytometry (Beckton Dickinson FACS CANTO II flow cytometer, equipped with FACSDiva 8.0), applying a staining protocol, as previously reported (Scarsi M, Zanotti C, Chiarini M, Imberti L, Piantoni S, Frassi M, Tincani A, Airò P. Reduction of peripheral blood T cells producing IFN-γ and IL-17 after therapy with abatacept for rheumatoid arthritis. Clin Exp Rheumatol. 2014 Mar-Apr;32(2):204-10. Epub 2014 Jan 14. PMID: 24428959).

CD3+CD4+ T-cells and CD3+CD8+ T-cells (defined as CD3+CD4-) were detected in all patients. In overall T-cells and in each of the two groups we inquired the production of interferon-γ (INF-γ) and of interleukin-17 (IL-17).

*Cytokine production by T cells after in vitro activation.*

Peripheral blood mononuclear cells obtained by Ficoll-Paque gradient centrifugation were thawed, cultured at 37°C temperature, and stimulated with phorbol 12-myristate 13-acetate and ionomycin (Invitrogen, eBioscience Cell Stimulation Cocktail (500X), Thermofisher Scientific) in order to enhance the production of INF-γ and IL-17. After the stimulation, cultured cells remained at 37°C and 5% CO_2_ pressure for 1.5 hours.

Cells were washed and stained for 30 minutes at room temperature with V450 anti-CD3 (Beckton Dickinson) and APC anti-CD4 (Beckton Dickinson), for the identification of T cells subsets. Cells were fixed and permeabilized (Cytofix/Cytoperm fixation/permeabilization kit, Beckton Dickinson) for 20 minutes. Intra-cytoplasmic staining was performed for 20 minutes using PE anti-IFN-γ (Beckman Coulter) and Alexa Fluor 488 anti-IL-17A (Beckton Dickinson). Cells were analyzed at the cytometer within 2 hours after the staining. Data were visualized using FlowJo (FlowJo LLC, Ashland, OR/version 10; https://www.flowjo.com).

Figure S1. Study flow diagram. The figure displays diagram of study and reasons of exclusion of splenectomized patients. Subsequent an age- and sex-matching was performed among cholecystectomized patients to compose the control group. Visits were randomly scheduled. Personnel aware of type of prior surgical intervention collected medical history, chronic treatment and laboratory values, while all other procedures and evaluations were performed by operators unaware of patients’ group. ABPM = ambulatory blood pressure monitoring; AO = adaptive optics.

Figure S2. Red blood cells in cases and controls. Microscopy evaluation showed the presence of >10% pitted red blood cells (white arrows) among patients underwent splenectomy (panel A and B), confirming the functional asplenism in these patients. Conversely, all cholecystectomized subjects had <5% of pitted red blood cells (panel C and D).

**Figure S3.** Combinations of anti-hypertensive drugs. The UpSet plot displays any combination of anti-hypertensive drugs of patients diagnosed for hypertension in splenectomy group (upper panel) and in cholecystectomy group (lower panel). Vertical bars above correspond to the number of patients with a specific combination, horizontal bars on the left correspond to the total number of patients taking each specific drug type. arb = angiotensin receptor blocker; acei = angiotensin-converting enzyme inhibitor; bb = β-blocker; ccb = calcium-channel antagonist.

Figure S4. Cumulative incidence of new diagnosis of hypertension. Kaplan-Meier curves show the rate of new diagnosis of hypertension in patients that underwent either splenectomy or cholecystectomy and were not diagnosed for hypertension at the time of surgical intervention.

**Figure S1.**

**
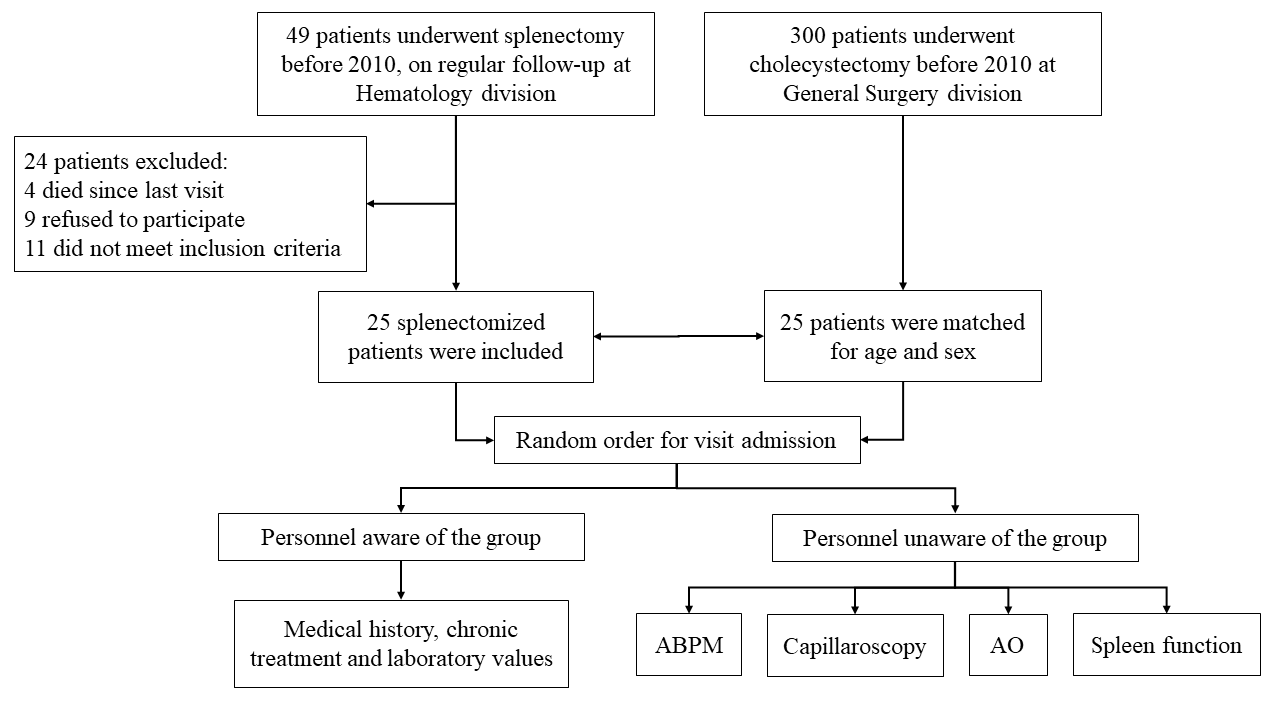
**

**Figure S2.**

**
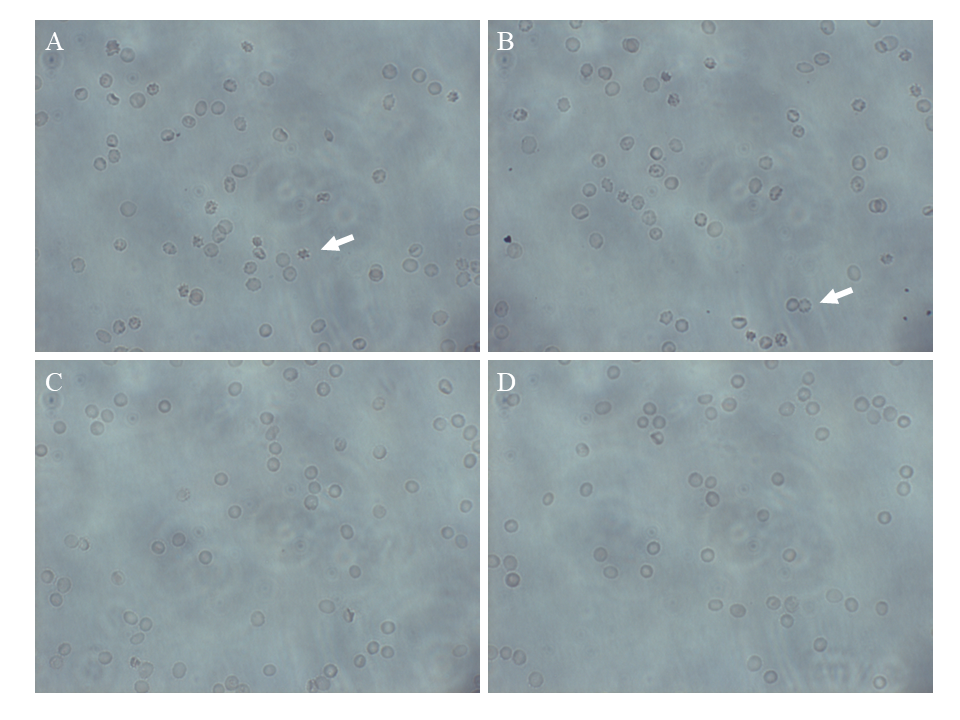
**

**Figure S3.**


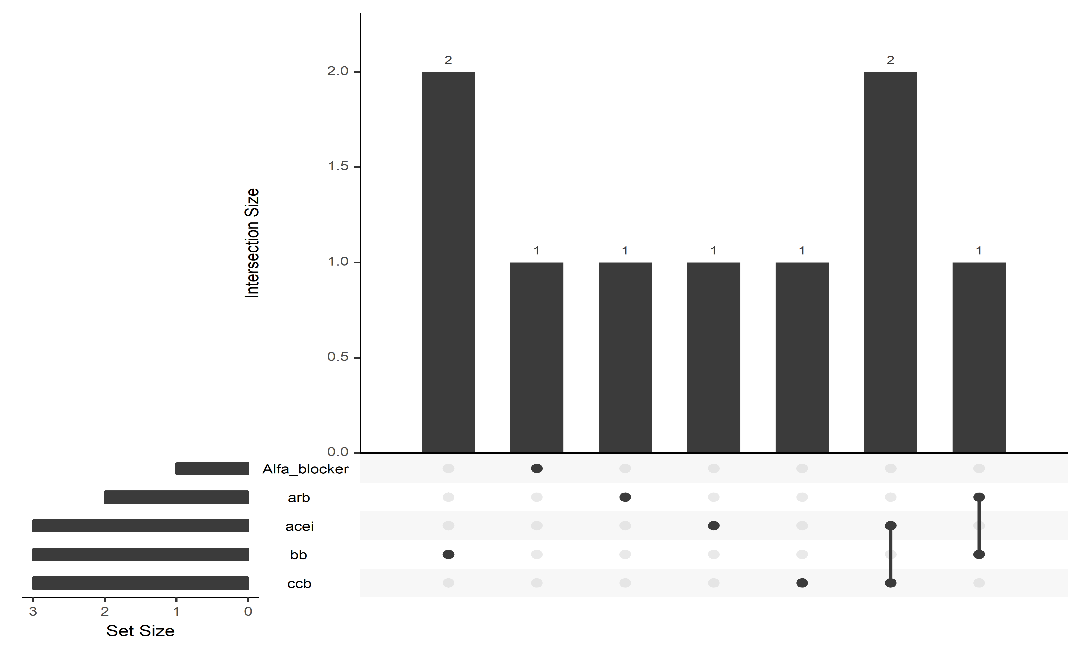

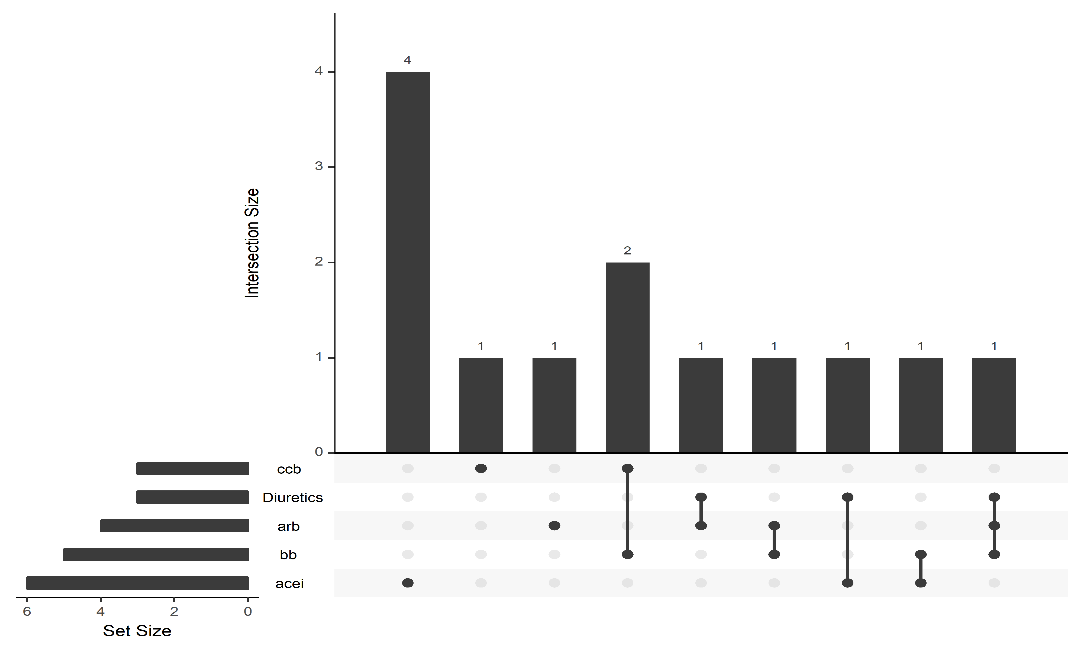


**Figure S4.**


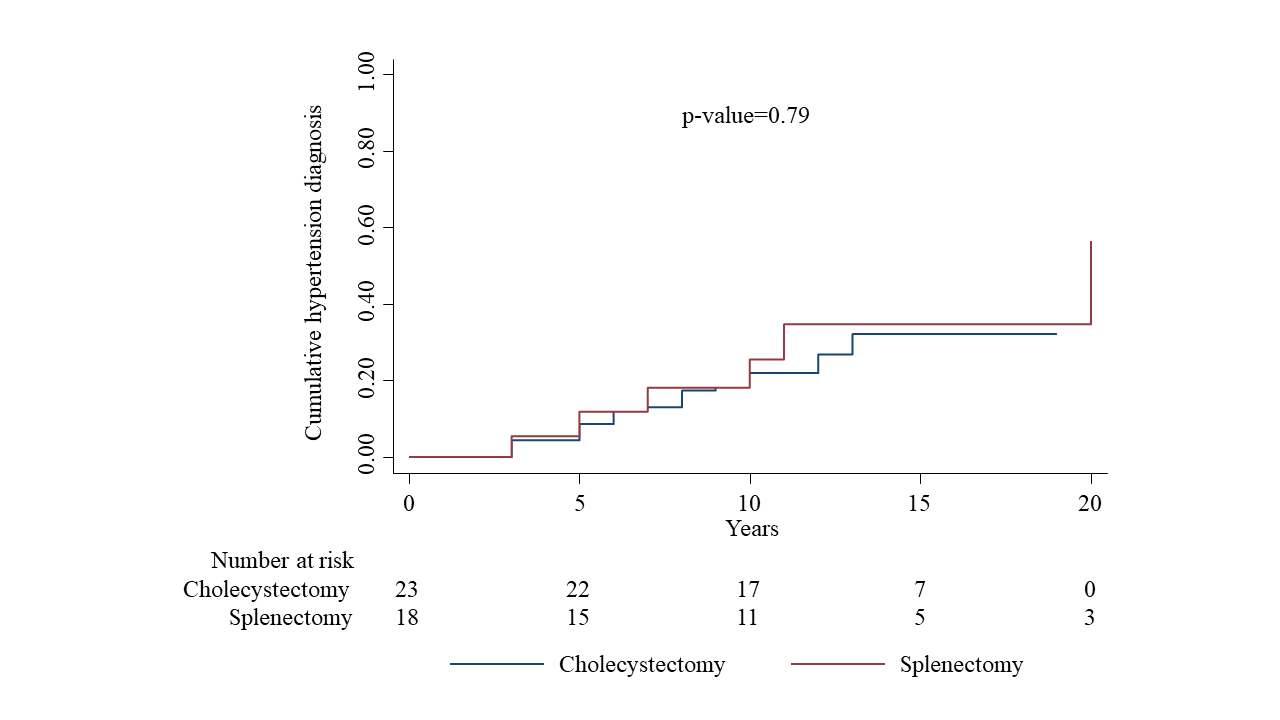


**Table S1. Linear regressions between wall cross-sectional area, wall-to-lumen ratio, dorsal and forearm capillary recruitment, and main values provided by 24h-ambulatory blood pressure monitoring**

|  | Overall (n=50) | | | Splenectomy (n=25) | | Cholecistectomy (n=25) | | p_interation_ |
| --- | --- | --- | --- | --- | --- | --- | --- | --- |
|  | β-coeff | 95% CI | p-value | β-coeff | 95% CI | β-coeff | 95% CI |  |
| WCSA vs. |  |  |  |  |  |  |  |  |
| Systolic BP 24h | -0.4 | -30.6;29.8 | 0.98 | 0.08 | -43.9;59.4 | -0.08 | -45.6;32.5 | 0.61 |
| Diastolic BP 24h | -7.0 | -50.0;36.1 | 0.75 | 0.43 | -9.6;180 | -0.25 | -76.9;21.2 | 0.019 |
| Mean BP 24h | -3.9 | -45.9;38.1 | 0.85 | 0.36 | -29;176.3 | -0.17 | -63;28.9 | 0.066 |
| AIx@75 | -15.0 | -51.3;21.4 | 0.41 | -0.14 | -81.6;47.6 | -0.1 | -56.7;35.7 | 0.85 |
|  |  |  |  |  |  |  |  |  |
| WLR vs. |  |  |  |  |  |  |  |  |
| Systolic BP 24h | 0.001 | -0.01;0.01 | 0.24 | -0.09 | -0-003;0.002 | 0.08 | -0.002;0.003 | 0.83 |
| Diastolic BP 24h | 0.001 | -0.01;0.01 | 0.30 | 0.1 | -0.004;0.006 | 0.2 | -0.002;0.006 | 0.55 |
| Mean BP 24h | -0.001 | -0.01;0.01 | 0.54 | -0.006 | -0.005;0.005 | 0.22 | -0.002;0.005 | 0.59 |
| AIx@75 | -0.001 | -0.01;0.01 | 0.52 | -0.03 | -0-003;0.003 | -0.18 | -0.004;0.002 | 0.56 |
|  |  |  |  |  |  |  |  |  |
| Dorsal recruitment vs. |  |  |  |  |  |  |  |  |
| Systolic BP 24h | -0.2 | -0.52;0.13 | 0.24 | -0.01 | -0.41;0.39 | -0.3 | -0.93;0.2 | 0.23 |
| Diastolic BP 24h | -0.15 | -0.62; 0.32 | 0.53 | -0.21 | -1.1;0.4 | -0.13 | -0.94;0.52 | 0.80 |
| Mean BP 24h | -0.24 | -0.68;0.19 | 0.27 | -0.12 | -0.86;0.50 | -0.25 | -1.03;0.28 | 0.68 |
| AIx@75 | 0.2 | -0.17;0.61 | 0.28 | -0.01 | -0.50;0.49 | 0.23 | -0.3;0.98 | 0.37 |
|  |  |  |  |  |  |  |  |  |
| Forearm recruitment vs. |  |  |  |  |  |  |  |  |
| Systolic BP 24h | -0.12 | -0.46;0.22 | 0.48 | -0.13 | -0.68;0.37 | -0.08 | -0.56;0.39 | 0.83 |
| Diastolic BP 24h | 0.04 | -0.47;0.55 | 0.87 | 0.36 | -0.15;1.94 | -0.25 | -0.97;0.26 | 0.02 |
| Mean BP 24h | -0.08 | -0.57;0.41 | 0.74 | 0.13 | -0.76;1.36 | -0.2 | -0.84;0.30 | 0.29 |
| AIx@75 | -0.01 | -0.44;0.42 | 0.95 | -0.02 | -0.77;0.70 | -0.01 | -0.56;0.56 | 0.93 |
|  |  |  |  |  |  |  |  |  |
| PWV vs. |  |  |  |  |  |  |  |  |
| Systolic BP 24h | 0.090 | 0.28;0.15 | 0.006 | 0.14 | 0.01;0.28 | 0.81 | 0.005;0.15 | 0.3419 |
| Diastolic BP 24h | 0.056 | -0.03;0.14 | 0.203 | 0.02 | -0.21;0.26 | 0.11 | -0.0057;0.224 | 0.4166 |
| Mean BP 24h | 0.070 | -0.01;0.15 | 0.087 | 0.11 | -0.11;0.33 | 0.088 | -0.013;0.190 | 0.8034 |
| AIx@75 | -0.09 | -0.16;-0.01 | 0.026 | -0.10 | -0.23;0.036 | -0.08 | -0.18;0.022 | 0.8020 |

WCSA = wall cross-sectional area; BP = blood pressure; WLR = wall-to-lumen ratio; AIx@75 = augmentation index standardized at hear rate of 75 beat per minute; PWV= pulse wave velocity.
